# *Lactobacillus brevis* Alleviates DSS-Induced Colitis by Reprograming Intestinal Microbiota and Influencing Serum Metabolome in Murine Model

**DOI:** 10.3389/fphys.2019.01152

**Published:** 2019-09-18

**Authors:** Sujuan Ding, Yong Ma, Gang Liu, Wenxin Yan, Hongmei Jiang, Jun Fang

**Affiliations:** ^1^College of Bioscience and Biotechnology, Hunan Agricultural University, Changsha, China; ^2^Hunan Provincial Key Laboratory of Animal Nutritional Physiology and Metabolic Process, CAS Key Laboratory of Agro-Ecological Processes in Subtropical Region, Institute of Subtropical Agriculture, Chinese Academy of Sciences, National Engineering Laboratory for Pollution Control and Waste Utilization in Livestock and Poultry Production, Changsha, China

**Keywords:** colitis, murine, *Lactobacillus brevis*, intestinal microbiota, metabolome

## Abstract

The aim of this study was to examine the effects of *Lactobacillus brevis* on the microbial community and serum metabolome in colitis induced by dextran sulfate sodium (DSS). ICR mice were randomly distributed into three treatment groups: (i) *L. brevis* treatment alone (control), (ii) DSS administration alone, and (iii) treatment with *L. brevis* and DSS. Our results demonstrate that *L. brevis* treatment significantly alleviated DSS-induced body weight loss and colon inflammation. In addition, LC-MS analysis of serum metabolites revealed that *L. brevis* treatment increased the serum level of metabolites against inflammatory responses or oxidative stressors caused by DSS in the murine model. By detecting colonic microbiota, *L. brevis* increased colonic microbial diversity after challenging with DSS, and increased the relative abundance of Alloprevotella at genus, but Bacteroidales was reduced (*P* < 0.05). These result indicated that *L. brevis* could lower the severity of colitis induced by DSS via improving reprogramming the serum metabolome and intestinal microbiota. These findings suggest that the probiotic *L. brevis* may prevent tissue damage from colitis.

## Introduction

The regression of mucosal inflammation promotes the recovery of the epithelial barrier and proper tissue repair. It also restores normal organ function and steady-state microenvironment conditions (Leoni et al., 2015; Lopetuso et al., 2018). The pathogenesis of inflammatory bowel disease (IBD) is associated with dysfunction and a delay in mucosal healing (Neurath and Travis, 2012). Ulcerative colitis (UC) is a chronic IBD with unknown origin (Ng et al., 2013). Its recurrence and regression can severely affect patients’ quality of life. Despite efforts to reveal its pathogenesis and develop new therapies, UC remains a challenge for the medical and scientific communities (Detel et al., 2016).

Probiotics are live microorganisms that, when administered in adequate amounts, confer health benefits on the host (Hill et al., 2014). Indeed, it has long been proposed that the consumption of lactic acid bacteria in fermented products can improve health and longevity in humans (Martin et al., 2017). The bacteria in the gut constantly interact with human cells in a variety of ways (Sekirov et al., 2010). It is widely established that the intestinal microbiota can regulate epithelial function, prevent pathogenic bacteria colonization, and affect immune responses (Martin et al., 2017). Several studies have revealed that *Lactobacillus brevis* KB290 can reduce the severity of colitis in murine models by increasing the ratio of CD11c^+^ MP dendritic cells to CD103^–^ dendritic cells in the colon (Fuke et al., 2018). Studies have also shown that *L. brevis* SBC8803 can regulate the expression of tumor necrosis factor α and interleukins 1b and 12 and improve the barrier function of the intestinal epithelium under oxidative stress. These results indicate that *L. brevis* SBC8803 contributes to the maintenance of intestinal homeostasis and can alleviate intestinal inflammation (Ueno et al., 2011). Moreover, a study has found that polyphosphate, an active substance from *L. brevis*, can down-regulate the expression of inflammatory and fibrosis-related molecules in intestinal epithelial cells to prevent inflammation and fibrosis (Kashima et al., 2015).

Metabolomic analysis is emerging as a powerful approach in system biology research because it provides unique insights into the understanding of organisms, disease diagnosis, pathology, and toxicology. Metabolomics can reveal the type of ongoing cellular processes in the organism by analyzing the chemical fingerprints of cells (Xu et al., 2012; Weng et al., 2015). For example, metabolomics analyses of serum samples from children with Crohn’s disease (CD) and UC have revealed that most of the chemically annotated metabolites belonged to the phospholipids and that they were reduced in CD and UC patients relative to healthy individuals subjects (Daniluk et al., 2019). In addition, the partial least-squares discriminant analysis (PLS-DA) load map of murine models of dextran sulfate sodium (DSS)-induced colitis showed that succinic acid, indole-3-acetic acid, glutamic acid, and glutamine are the main metabolites that separate the stages of colitis (Shiomi et al., 2011).

Currently, most probiotic strains are Lactobacilli and other lactic acid bacteria or Bifidobacteria (de Vrese and Schrezenmeir, 2008). There is mounting evidence that Lactobacillus can counteract intestinal inflammation, but the specific mechanism for such protection is unclear (Sung and Park, 2013). DSS-induced colitis in model animals has been shown to anthropic UC in diverse disease pathologies, ranging from disordered immune responses to clinical manifestations (Detel et al., 2016). Here, using a murine model, we aimed to investigate the effects of *L. brevis* on the severity of DSS-induced colitis and changes in the gut microbiota and the serum metabolome and to reveal the correlation between the intestinal microbiota and serum metabolites.

## Materials and Methods

### Bacteria Preparation

*Lactobacillus brevis* stored in the Chinese Academy of Sciences Key Laboratory of Agro-ecological Processes in Subtropical Region (Changsha, China) were incubated in MRS broth (De Man, Rogosa, Sharpe) at 37°C for 24 h. Cultured bacterial fluid was cultured in MRS agar medium for 24 h at 37°C. Colonies were quantified, and the number of colonies per mL was determined. The bacteria samples were centrifuged at 5000 rpm for 10 min at 4°C. The pellet was resuspended in sterile normal saline solution at 2 to 5 × 10^10^ CFU/mL.

### Animal and Experimental Design

All animal procedures were approved by the Animal Ethics Committee of Hunan Agricultural University. Eight-week-old female ICR mice were purchased from SLAC Laboratory Animal Central (Changsha, China). DSS was purchased from Dalian Meilun Biotech., Co., Ltd. (M.W: 36000–50000, Dalian, China). All mice were housed in a pathogen-free mouse colony in a standard environment (temperature, 23°C ± 1°C; humidity, 50 ± 10%; 12-h alternating lighting cycles). After a 7-day adaptation period, the mice were randomly assigned to three groups of eight. The first group was treated with *L. brevis* (control), the second group was treated with 5% DSS (DSS), and the third group was treated with *L. brevis* and 5% DSS (LB-DSS). All mice had unlimited access to the basal diet feed and water. Our study lasted for a total of 19 days, the mice was administrated via intragastric administration with *L. brevis* or sterile saline, then DSS-treated group was given with 5% DSS in water from days 7 to 12, and continued to treat with *L. brevis* or sterile saline (12–19 days). At the end of the 19 days, the mice underwent 8 h of fasting, and blood samples were collected from the orbital blood vessels. The mice were then killed, and the colon lengths were measured. The middle part of the colon was then fixed using 4% formaldehyde. The colon contents were collected, frozen in liquid nitrogen, and stored at −80°C. The final weight of the mice was recorded.

### Colonic Histopathology

Colon samples were fixed using 4% formaldehyde, dehydrated using ethanol gradient, and embedded in paraffin, and 8-μm sections were stained with hematoxylin and eosin and viewed with an Olympus BX41 microscope (Münster, Germany).

Colon tissue samples were examined and graded according to the severity of inflammation as previously described (Vukelic et al., 2018). Histological examination showed that each colon tissue specimen was graded according to the severity of inflammation (no, 0; mild, 1; moderate, 2; severe, 3); inflammatory cell infiltration (normal, 0; mucosa, 1; mucosa with submucosa, 2; transmural extension of infiltration, 3); epithelial lesions (intact, 0; deformation of crypt structure, 1; erosion, 2; ulcer, 3); the degree of lesion (none, 0; point, 1; multifocal, 2; diffuse, 3); edema score (no, 0; mild edema of mucosa, 1; submucosa and mucosa, 2; whole wall of colon, 3). The final histologic injury score for the colon was the sum of the individual scores.

### Serum Metabolomic Analyses

Serum samples were thawed slowly on ice. A 100-μL serum aliquot was added to 300 μL of methanol (Merck, Darmstadt, Germany) and 10 μL of 2.8 mg/mL 2-Chloro-L-phenylalanine as internal standard (Sigma, St. Louis, MO). The mixture was briefly shaken for 30 s using a vortex and kept at −20°C for 1 h. They were then centrifuged at 12,000 rpm for 10 min at 4°C; and 200-μL aliquots of each of the sample supernatant were transferred into sampling bottles for analysis. Each sample was analyzed using a LC-MS analysis platform (Thermo Fisher Scientific, Ultimate 3000LC, Q Exactive) and a Hyper gold C_18_ (3 μm, 100 × 4.6 mm) column (Waters, Dublin, Ireland). The column temperature was maintained at 40°C and the flow rate at 0.35 mL/min. The automatic injector temperature was 4°C and the injection volume was 10 μL. The mobile phase was composed of 5% acetonitrile and 0.1% formic acid (A) (Merck) and acetonitrile with 0.1% formic acid (B). Gradient elution of the mobile phase is shown below: 0/5, 1/5, 2/40, 7/80, 11/95, 15.01/95, 15.5/5 and 19.5/5 (min/%B). The MS parameters were as follows, ESI+: Heater Temp = 300°C, Sheath Gas Flow rate = 45 arb, Aux Gas Flow Rate = 15 arb, Sweep Gas Flow Rate = 1 arb, spray voltage = 3.0 kV, Capillary Temp = 350°C, S-Lens RF Level = 30%; ESI–: Heater Temp = 300°C, Sheath Gas Flow rate = 45 arb, Aux Gas Flow Rate = 15 arb, Sweep Gas Flow Rate = 1 arb, spray voltage = 3.2 kV, Capillary Temp = 350°C, S-Lens RF Level = 60%. Data were extracted and analyzed according to a previous study (Ding et al., 2019).

### 16S Ribosomal RNA Amplicon Sequencing

Fecal samples were prepared for the MiSeq Library, followed by Illumina MiSeq 2 × 300 bp high throughput sequencing and bioinformatics analysis. Briefly, the cDNA library was constructed using a two-step PCR amplification method. Firstly, the target fragment was amplified using specific primers (inner primers) and recovered by glue. Then, the recovered product was used as the template for secondary PCR amplification (outer primers). The inner primer was F-(5′-TTC CCT ACA CGA CGC TCT TCC GAT CT-specific primer-3′) and R-(5′-GAG TTC CTT GGC ACC CGA GAA TTC CA- specific primer -3′), and the outer primers was F-(5′-AAT GAT ACG GCG ACC ACC GAG ATC TAC AC- barcode – TCT TTC CCT ACA CGA CGC TC -3′) and R-(5′-CAA GCA GAA GAC GGC ATA CGA GAT- barcode – GTG ACT GGA GTT CCT TGG CAC CCG AGA-3′). All PCR products were recovered using AxyPrepDNA gel recovery kit (AXYGEN, Hangzhou, China), and quantitative analysis was conducted using FTC-3000 real-time PCR. The samples were mixed according to the molar ratio, and the library was prepared for standard Illumina sequencing using HiSeq2500 PE250 (Illumina, United States).

### Correlation Analysis Between Serum Differential Metabolites and Colonic Microbiota

To explore the relationship between serum metabolic profiles and colonic microbiota in the development of DSS-induced colitis, Pearson correlation analysis was carried out using GraphPad Prism 7.00 for Windows software. Statistical significance was defined by a *P*-value of less than 0.05.

### Data Analysis

All quantifications underwent analysis of variance to test the homogeneity of variance by Levene’s test and Student’s *t*-test (SPSS 21 software). Statistical significance was defined by a *P*-value of less than 0.05.

## Results

### *Lactobacillus brevis* Counteracts DSS-Mediated Weight Loss and Colonic Injury in Mice

**FIGURE 1:**
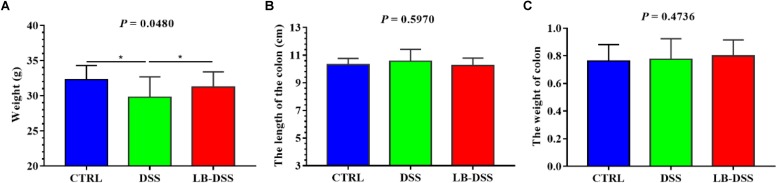
Effects of *L. brevis* on DSS-induced colitis. **(A)** Final weight of the control, DSS, and LB-DSS mice. **(B)** Length of the colon of control, DSS, and LB-DSS mice. **(C)** Weight of the colon of control, DSS, and LB-DSS mice. ^∗^*P* < 0.05.

The mice were treated with or without *L. brevis* for 7 days, and then given 5% DSS for 5 days, followed by *L. brevis* treatment for another 7 days. The final animal weight, colon weight, and colon length were quantified (Figure 1). We observed that the mean weight of DSS mice was lower than those of control and LB-DSS mice (*P* ≤ 0.05), indicating that DSS treatment reduced overall body weight, and that *L. brevis* treatment counteracted DSS-mediated body weight loss. In addition, we observed no significant differences between the mean weights of control and LB-DSS mice, indicating that *L. brevis* did not affect the homeostatic body weight in the absence of DSS.

### *Lactobacillus brevis* Retards DSS-Induced Colitis

**FIGURE 2:**
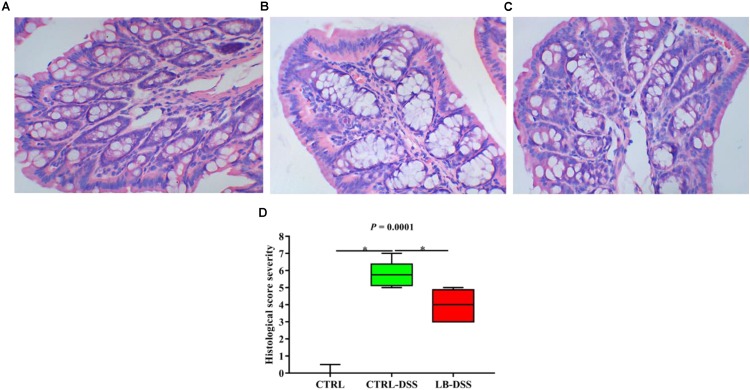
Severity of colon tissue inflammation in mice given with DSS. Effects of *L. brevis* on colonic histology (**A:** control; **B:** DSS; **C:** LB-DSS), and corresponding histologic severity scores **(D)**. ^∗^*P* < 0.05.

Histologic examination showed multiple erosive lesions and extensive inflammatory cell infiltration in the colon tissue of mice given with DSS. Infiltrated cells mainly consisted of macrophages, lymphocytes, neutrophils, and occasional eosinophils (Figure 2B). Intuitively, no lesions were observed in the colon tissue of control mice (Figure 2A). Although the colon tissue of LB-DSS mice showed some inflammatory lesions (Figure 2C), the severity of inflammation was less than that in the DSS mice (*P* ≤ 0.05; Figure 2D).

### *Lactobacillus brevis* Affects the Serum Metabolomic Profiles During Colitis

**FIGURE 3:**
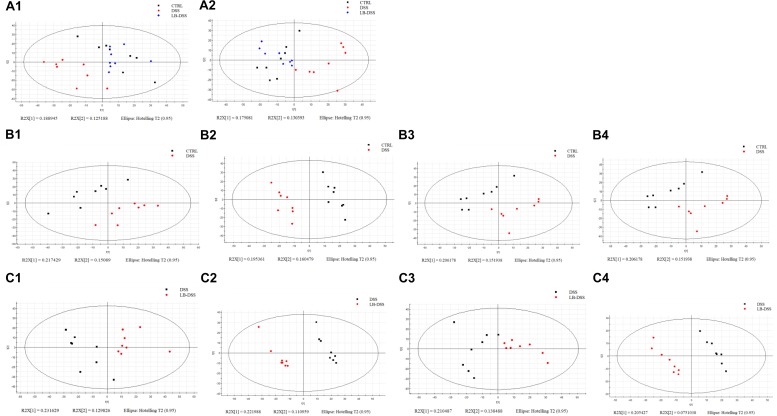
Score plots of principal component analysis (PCA) and OPLS-DA models with their corresponding R2X and T2 values. PCA score plots among the three groups in positive ion mode **(A1)** or negative ion mode **(A2)**, PCA **(B1,C1)** and PLS-DA **(B2,C2)** score plots between two groups in positive mode, and PCA **(B3,C3)** and PLS-DA **(B4,C4)** score plots between two groups in negative mode.

Principal component analysis (PCA) was used to determine the intrinsic similarity of spectral profiles (Figures 3B3,C3). Each scatterplot displays a serum sample in positive ion model (Figures 3A1,B1,C1) and negative ion model (Figures 3A2,B2,C2). This analysis was performed by Umetrics (Sweden). To reveal significant differences in metabolite abundance, two-group comparisons of the samples were conducted and analyzed using supervised multidimensional statistical method PLS-DA (Figures 3B2,B4,C2,C4). The variable importance in the projection (VIP > 1.5) of PLS-DA mode and *P*-values were used to identify metabolites that exhibited differential abundances (Table 1). A total of 22 metabolites were obtained, including 20 in the control vs. DSS comparison and 14 in the DSS vs. LB-DSS comparison. We found that the abundances of 2-hydroxyglutarate, epinephrine, oxalacetic acid, pyridine, guanosine, 6-methylthioguanosine monophosphate, N1-acetylspermidine, and ascorbic acid exhibited opposite trends in the control vs. DSS (increase) and DSS vs. LB-DSS (decrease) comparisons (Table 1). We found that the abundances of cholesteryl acetate, tetrahydrocortisone, carnosic acid, and N-undecanoylglycine also exhibited opposite trends in the control vs. DSS (decrease) and DSS vs. LB-DSS (increase) comparisons (Table 1). In addition, we observed some unique metabolites, such as serotonin, 11-dehydrocorticosterone, and indole (relative increase) and vitamin A2, gamma-linolenic acid, glycochenodeoxycholate-3-sulfate, arachidonic acid, and docosahexaenoic acid (relative decrease) in the control vs. DSS comparison. Lastly, two metabolites (7α-hydroxy-3-oxo-4-cholestenoate and 25,26-dihydroxyvitamin D) exhibited relative increases in the DSS vs. LB-DSS comparison.

**TABLE 1:** Metabolomic changes in serum of control, DSS, and LB-DSS mice.

**Metabolites**	**Calc m/z**	**Control vs. DSS**				**DSS vs. LB-DSS**			
			
		**(Log) FC**	**VIP**	***P*-value**	**Change**	**(Log) FC**	**VIP**	***P*-value**	**Change**
Cholesteryl acetate	429.3727	–1.55	1.87	0.00016	↓	1.58	1.73	0.00027	↑
Serotonin	177.1022	1.10	1.52	0.00640	↑	–	–	–	–
Vitamin A2	285.2213	–1.22	2.01	0.00002	↓	–	–	–	–
Gamma-linolenic acid	243.2119	–1.82	1.84	0.00026	↓	–	–	–	–
2-Hydroxyglutarate	149.0444	1.41	1.85	0.00023	↑	–1.63	1.88	0.00004	↓
Epinephrine	184.0968	1.12	1.93	0.00006	↑	–1.18	1.70	0.00043	↓
Tetrahydrocortisone	365.2323	–3.85	1.93	0.00007	↓	2.74	1.67	0.00058	↑
Oxalacetic acid	133.0131	1.09	2.01	0.00001	↑	–1.54	2.05	0.00000	↓
Glycochenodeoxycholate-3-sulfate	530.2782	–2.71	1.99	0.00002	↓	–	–	–	–
Pyridine	80.0495	1.85	1.74	0.00136	↑	–1.81	1.62	0.00128	↓
7α-Hydroxy-3-oxo-4-cholestenoate	431.3156	–	–	–	–	–1.28	1.58	0.00166	↓
Arachidonic acid	305.2475	–1.46	1.85	0.00024	↓	–	–	–	–
Guanosine	284.0989	1.24	1.55	0.00487	↑	–1.43	1.51	0.00521	↓
Carnosic acid	297.1861	–4.04	1.96	0.00004	↓	2.81	1.69	0.00046	↑
Docosahexaenoic acid	329.2475	–1.47	1.89	0.00014	↓	–	–	–	–
6-Methylthioguanosine monophosphate	394.0581	1.52	1.51	0.00653	↑	–1.63	1.66	0.00069	↓
N-Undecanoylglycine	244.1907	–1.39	1.76	0.00066	↓	1.22	1.65	0.00074	↑
N1-Acetylspermidine	188.1757	1.58	1.83	0.00028	↑	–1.73	1.78	0.00012	↓
11-Dehydrocorticosterone	345.206	1.56	1.64	0.00201	↑	–	–	–	–
25,26-dihydroxyvitamin D	417.3363	–	–	–	–	–1.33	1.53	0.00320	↓
Indole	82.0451	–1.35	2.01	0.00002	↓	–	–	–	–
Ascorbic acid	177.0394	1.52	1.81	0.00036	↑	–1.72	1.94	0.00001	↓
N4-Acetylcytidine	286.1034	–	–	–	–	1.06	1.74	0.00059	↑

*Control vs. DSS, the control group compared with the DSS group; DSS vs. LB-DSS, the DSS group compared with LB-DSS group. FC, fold change (the ratio of mean metabolite levels between the groups), (Log) FC > 0 indicated a relatively higher concentration in the control or DSS group, while (Log) FC < 0 indicated a relatively lower concentration in the control or DSS group. VIP, variable importance in the projection; **↑/↓**, increase/decrease, –, no changes. Significant threshold was set at VIP > 1.50 and *P* < 0.05.*

### *Lactobacillus brevis* Affects the Alpha Diversity in the Murine Colon During Colitis

**FIGURE 4:**
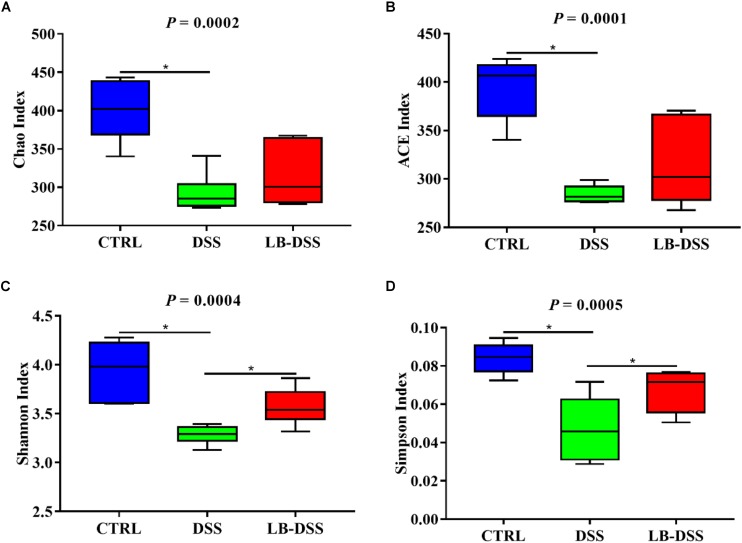
Diversity indexes of microbiota in the mouse colon. Vox plots depict the Chao index **(A)**, ACE index **(B)**, Shannon index **(C)**, and Simpson index **(D)** of the colonic microbiota of the control, DSS, and LB-DSS mice. ^∗^*P* < 0.05.

The 16S rRNA in v3-v4 region extracted from the colonic samples were analyzed. The colonic microbiota diversity was determined using the Chao index, ACE index, Shannon index, and Simpson index (Figures 4A–D). We found that the colonic microbiota diversity decreased in DSS mice compared with the control mice. Importantly, LB-DSS mice exhibited higher Shannon and Simpson indices than the DSS mice (*P* ≤ 0.05) (Figures 4C,D). Albeit insignificant, similar trends were observed for the Chao and ACE indices (Figures 4A,B). These results indicated that *L. brevis* prevented the loss of microbial diversity in the mouse colon after DSS challenge.

### *Lactobacillus brevis* Affects Microbial Abundance at the Phylum Level During Colitis

**FIGURE 5:**
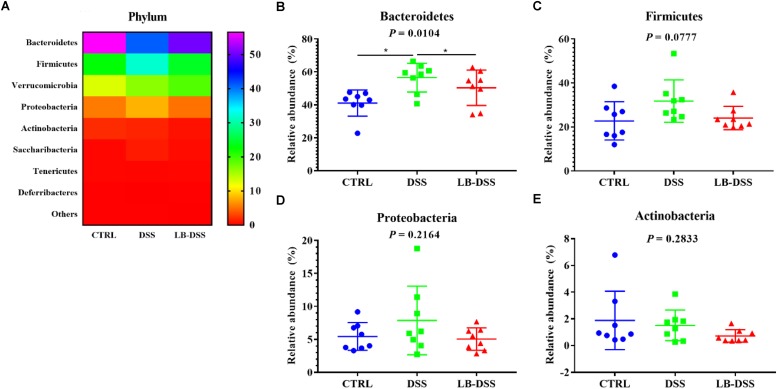
Analysis of the microbial composition at the phylum level. **(A)** Relative abundances of microbial phyla in the mouse colon. Comparisons of the relative abundances of Bacteroidetes **(B)**, Firmicutes **(C)**, Proteobacteria **(D)**, and Actinobacterial **(E)** in the colon of control, DSS, and LB-DSS mice. ^∗^*P* < 0.05.

The dominant bacterial phyla observed in the mouse colon were Bacteroidetes, Firmicutes, Verrucomicrobia and Proteobacteria. They accounted for more than 97% of the microbiota. The proportion of Bacteroidetes was 41.09, 56.53, and 50.36%, that of Firmicutes was 22.75, 31.78, and 24.09%, that of Verrucomicrobia was 12.86, 16.45, and 19.02%, and that of Proteobacteria was 5.44, 7.87, and 5.04% in the control group, DSS group, and LB-DSS group, respectively (Figure 5A). Moreover, the relative abundance of Bacteroidetes was increased in the DSS group compared with the other two groups (Figure 5B), but there are no other differences (Figures 5C–E).

### *Lactobacillus brevis* Affects Microbial Abundance at the Order During Colitis

**FIGURE 6:**
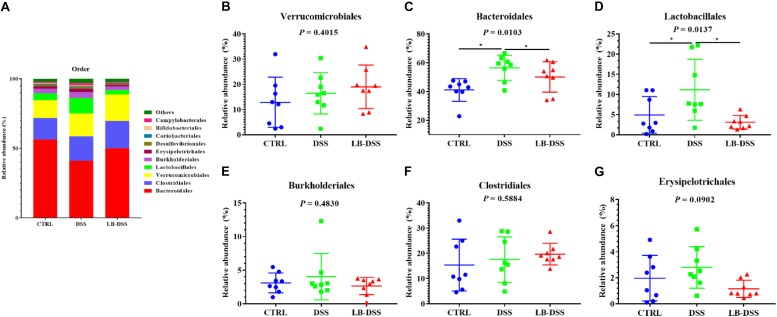
Analysis of the microbial composition at the order level. **(A)** Relative abundances of microbial orders in the mouse colon. Comparisons of the relative abundances of Verrucomicrobiales **(B)**, Bacteroidales **(C)**, Lactobacillales **(D)**, Burkholderiales **(E)**, Clostridiales **(F)**, and Erysipelotrichales **(G)** in the colon of control, DSS, and LB-DSS mice. ^∗^*P* < 0.05.

The top ten most abundant microbial orders are shown in Figure 6A. The three most abundant bacteria were Bacteroidales, Clostridiales, Verrucomicrobiales, and Lactobacillales. The proportion of Bacteroidales is 56.41, 41.07, and 50.07%, that of Clostridiales was 15.38, 17.58, and 19.69%, that of Verrucomicrobiales is 12.86, 16.45, and 19.02%, that of Lactobacillales is 4.93, 11.16, and 3.10% in the control group, DSS group, and LB-DSS group, respectively (Figure 6A). Moreover, the relative abundances of Bacteroidales (Figure 6C) and Lactobacillales (Figure 6D) were higher in the DSS group than in the other two groups (*P* ≤ 0.05). And there are no other differences (Figures 6B,E–G).

### *Lactobacillus brevis* Affects the Abundance of Microbial Genera During Colitis

**FIGURE 7:**
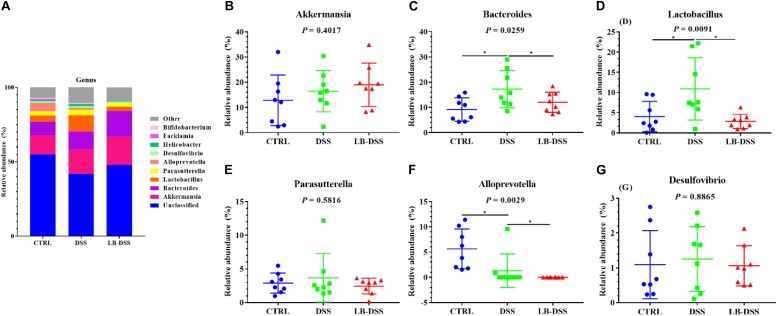
Analysis of the microbial composition at the genus level. **(A)** Relative abundances of microbial genera in the mouse colon. Comparisons of relative abundance of *Akkermansia* **(B)**, *Bacteroides* **(C)**, *Lactobacillus* **(D)**, *Parasutterella* **(E)**, *Alloprevotella* **(F)**, and *Desulfovibrio* **(G)** in the colon of control, DSS, and LB-DSS mice. ^∗^*P* < 0.05.

The top ten most abundant microbial genera were selected and made into bar percentage for analysis. The top three most abundant bacterial genera were found to be *Akkermansia*, *Bacteroides*, and *Lactobacillus*. The proportion of *Akkermansia* was 12.86, 16.45, and 19.02%, that of *Bacteroides* was 9.16, 12.05, and 17.23%, that of *Lactobacillus* was 4.06, 10.89, and 2.84% in the control group, DSS group, and LB-DSS group, respectively (Figure 7A). Moreover, the relative abundance of Lactobacillus was lower in the DSS group than in the other two groups (Figure 7D, *P* ≤ 0.05). The abundance of Alloprevotella was higher in the control group than in the DSS group and lower in the LB-DSS group than in the DSS group (Figure 7F, *P* ≤ 0.05). And there are no other differences (Figures 7B,C,E,G).

### Correlation Between Serum Metabolites and Colonic Microbiota

**FIGURE 8:**
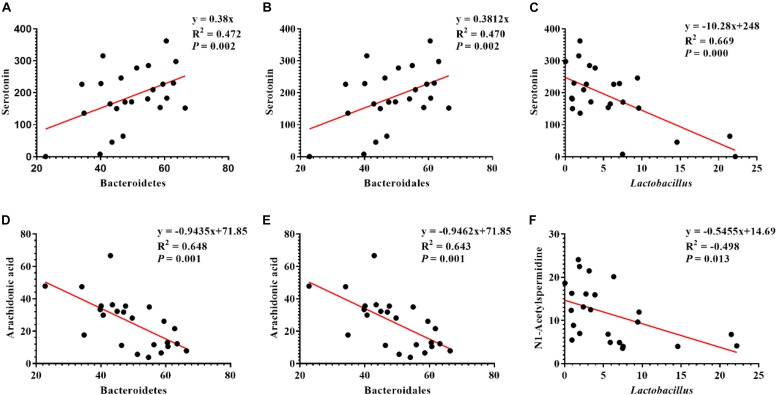
Correlation analysis of serum metabolites and colonic microbes. **(A)** Serotonin vs. Bacteroidetes, **(B)** serotonin vs. Bacteroidales, **(C)** serotonin vs. Lactobacillus, **(D)** arachidonic acid vs. Bacteroidetes, **(E)** arachidonic acid vs. Bacteroidales, and **(F)** N1-acetylspermidine vs. Lactobacillus.

Pearson correlation is one of the most commonly used statistical analysis method (Hannigan and Lynch, 2013). The results of Pearson correlation (*r*) between metabolites and microbiota are shown in Figure 8. We found different levels of correlation in six groups of correlation analyses (*P* < 0.05): serotonin vs. Bacteroidetes (*r* = 0.472; *P* = 0.002; Figure 8A), serotonin vs. Bacteroidales (*r* = 0.47; *P* = 0.002; Figure 8B), serotonin vs. *Lactobacillus* (*r* = 0.669; *P* = 0.000; Figure 8C), arachidonic acid vs. Bacteroidetes (*r* = 0.648; *P* = 0.001; Figure 8D), arachidonic acid vs. Bacteroidetes (*r* = 0.643; *P* = 0.001; Figure 8E), and N1-acetylspermidine vs. Lactobacillus (*r* = −0.498; *P* = 0.013; Figure 8F).

## Discussion

Inflammatory bowel disease patients are usually treated with anti-inflammatory, immunosuppressive, or biological agents, and some may even require surgery (Reid et al., 2011; Souza et al., 2015). Although these strategies can alleviate IBD symptoms, they do not provide permanent cure due to the cyclical and lifelong nature of IBD (Gareau et al., 2010). In recent years, an increasing body of evidence suggests that probiotics can prevent IBD in both experimental models and humans. In this study, our results reveal that *L. brevis* counteracted DSS-mediated body weight loss and inflammatory disruption of the colon in mice. In addition, *L. brevis* increased microbial diversity and the relative abundance of Bacteroidetes and Bacteroidales microbials in the colon after DSS challenge. In contrast, *L. brevis* treatment reduced the relative abundance of Alloprevotella microbials in the colon after DSS challenge. Metabolomic analysis showed that *L. brevis* treatment increased anti-inflammatory metabolites such as gamma-linolenic acid and carnosic acid and antioxidant metabolites such as ascorbic acid and 25,26-dihydroxyvitamin D in the serum.

The mammalian gastrointestinal tract is constantly exposed to a large number of bacteria, food, and environmental toxins (Kotula et al., 2014). The human intestinal mucosal surface is the largest body surface (∼200–300 m^2^) that is in constant contact with the external environment (Lievin-Le Moal, 2013). The intestinal epithelium is lined by a single layer of columnar epithelial cells and is folded into concave or crypts. These fully differentiated epithelial cells act as physical and functional barriers to defend the body against potentially harmful microbes and viruses within the gut microenvironment (Lievin-Le Moal and Servin, 2006). Studies have shown that DSS can reduce body weight, increase intestinal permeability, ulceration, inflammatory cell infiltration, and goblet cell loss in murine models (Gadaleta et al., 2011). Another study showed that *L. brevis* G-101 given at a dose of 1 × 10^8^ CFU/per mouse can prevent weight loss, colon shortening, and inflammation in ICR mouse models after trinitrobenzene sulfonic acid treatment (Jang et al., 2013). In addition, heat-killed body of *L. brevis* SBC8803 can alleviate DSS-induced intestinal tissue injuries and improve survival in mice (Ueno et al., 2011). Our results here suggest that *L. brevis* counteracted DSS-mediated body weight loss and colon inflammation and injury without affecting colon length and weight.

Many microbes have been found to have stably colonized in our intestines for decades, but their relative abundances vary with our diets (Faith et al., 2013; David et al., 2014). In mice and humans, the abundance of the bacterial community can be significantly altered within 24 h of dietary changes (Wu et al., 2011; Holmes et al., 2017). These findings provide new insights into the treatment of IBD because the intestinal microbiota plays an important role in intestinal inflammatory responses, including changes in the relative abundance and diversity of intestinal microbiota immunity (Lepage et al., 2011; Dubin et al., 2016). In the T5KO mouse model of adherent-invasive *Escherichia coli* (AIEC)-induced chronic colitis, AIEC invasion of the intestine is associated with the reduction of the overall microbiota diversity (Chassaing et al., 2014). Bacteroides is the dominant phylum in infectious colitis. It is the most abundant bacterial phylum in the healthy population, and the decline of its relative abundance is related to obesity and chronic diarrhea in humans (Eckburg et al., 2005; Turnbaugh et al., 2009; Khoruts et al., 2010). Culture-based studies have reported an increase in the abundance of Bacteroidetes in the colonic mucosa of IBD patients (Wu et al., 2013). Our results showed that *L. brevis* protected the diversity of colonic microbial community and decreased the relative abundance of Bacteroidetes after DSS treatment. We found a lower relative abundance of Lactobacillales in DSS mice than in control mice. This may be because the length of time *L. brevis* colonized the colon does not necessarily include the time when we collect colon contents. Therefore, even if *L. brevis* was given to the mice, it would not necessarily increase the relative abundance of Lactobacillales in the colon.

Metabolomic analysis provides data on all metabolic processes in cells and organisms (Shiomi et al., 2011). This quantitative analysis of metabolites is a promising method to identify biomarkers in IBD (Shiomi et al., 2011). In the control vs. DSS comparison, our results indicate that the concentrations of 2-hydroxyglutarate, epinephrine, oxalacetic acid, pyridine, guanosine, 6-methylthioguanosine monophosphate, N1-acetylspermidine, and ascorbic acid were higher and that those of cholesteryl acetate, tetrahydrocortisone, carnosic acid, and N-undecanoylglycine were lower. However, these changes exhibited opposite trends in the DSS vs. LB-DSS comparison. The N1-acetylspermidine metabolites, which are related to amino acid metabolism and oxidative stress in cells, are polyamines derived from ornithine and methionine and play an important role in cell membrane stability, biosynthesis of signaling molecules, and cell growth and differentiation (Ramot et al., 2010; Liu et al., 2015). Arachidonic acid is the main precursor of eicosanoid mediators, and the abundance of this metabolite is greatly increased after cell activation (Calder, 2011). Some metabolites are related to inflammation, such as ascorbic acid and guanosine (Sorice et al., 2014; Bellaver et al., 2015). Monoamine serotonin [5-hydroxytryptamine (5-HT)] is an important regulator of gastrointestinal tract and other organ systems and is a neurotransmitter in the brain (Yano et al., 2015).

In summary, our study provides insights into the mitigative role of *L. brevis* in colitis by regulating the colonic microbial community and altering the serum metabolome. Specifically, *L. brevis* retarded the manifestation of colitis, such as body weight loss and colonic tissue damage. *L. brevis* also improved the intestinal microorganism diversity, reduced the relative abundance of pathogenic bacteria, and altered the levels of serum metabolites. Our data indicate that the serotonin level was positively correlated with Bacteroidales and Lactobacillus abundances and negatively correlated with Lactobacillus abundance. The arachidonic acid level was negatively correlated with the abundances of Bacteroidetes and Bacteroidales, and the N1-acetylspermidine level was negatively correlated with the abundance of Lactobacillus. Serotonin plays an important role in gastrointestinal motility and intestinal dopaminergic neuron development, but its specific mechanism of influence is unknown. Taken together, our study demonstrates the feasibility and potency of using *L. brevis* in the treatment of IBD and provides a basis for further investigation regarding *L. brevis* and colitis.

## Data Availability

The data supporting this study can be found in NCBI using accession number SRP220500 (https://www.ncbi.nlm.nih.gov/sra/SRP220500).

## Ethics Statement

All animal procedures were approved by the Animal Ethics Committee of Hunan Agricultural University.

## Author Contributions

SD and YM performed the study and conducted data analysis. GL and JF designed the research. WY provided assistance for the study. SD and YM prepared the first draft of the manuscript. All authors read and revised the manuscript.

## Conflict of Interest Statement

The authors declare that the research was conducted in the absence of any commercial or financial relationships that could be construed as a potential conflict of interest.
